# Supplementary material for: A Critical View of the Application of the APEX Software (Aqueous Photochemistry of Environmentally-Occurring Xenobiotics) to Predict Photoreaction Kinetics in Surface Freshwaters
Source: Molecules. 2019 Dec 18;25(1):9. doi: 10.3390/molecules25010009 (PMC7017383; doi:10.3390/molecules25010009)
Supplement: Supplementary file 1 [file molecules-25-00009-s001.zip › 3.2.4_gcc-4.4.0.zip!/3.2.4_gcc-4.4.0/3.2.4_gcc-4.4.0/doc/octave/html/liboctave/Quadratic-Programming.html]

Quadratic Programming - Octave C++ Classes


Next: Nonlinear Programming,
Previous: Nonlinear Constraints,
Up: Optimization


---

### 9.5 Quadratic Programming

— : **QP** (void)  
— : **QP** (const ColumnVector &x, const Matrix &H)  
— : **QP** (const ColumnVector &x, const Matrix &H, const ColumnVector &c)  
— : **QP** (const ColumnVector &x, const Matrix &H, const Bounds &b)  
— : **QP** (const ColumnVector &x, const Matrix &H, const LinConst &lc)  
— : **QP** (const ColumnVector &x, const Matrix &H, const ColumnVector &c, const Bounds &b)  
— : **QP** (const ColumnVector &x, const Matrix &H, const ColumnVector &c, const LinConst &lc)  
— : **QP** (const ColumnVector &x, const Matrix &H, const Bounds &b, const LinConst &lc)  
— : **QP** (const ColumnVector &x, const Matrix &H, const ColumnVector &c, const Bounds &b, const LinConst &lc)

— : virtual ColumnVector **minimize** (void)  
— : virtual ColumnVector **minimize** (double &objf)  
— : virtual ColumnVector **minimize** (double &objf, int &inform)  
— : virtual ColumnVector **minimize** (double &objf, int &inform, ColumnVector &lambda) = 0;

— : virtual ColumnVector **minimize** (const ColumnVector &x)  
— : virtual ColumnVector **minimize** (const ColumnVector &x, double &objf)  
— : virtual ColumnVector **minimize** (const ColumnVector &x, double &objf, int &inform)  
— : virtual ColumnVector **minimize** (const ColumnVector &x, double &objf, int &inform, ColumnVector &lambda)

— : ColumnVector **minimize** (double &objf, int &inform, ColumnVector &lambda)
